# Cartilage Protective and Chondrogenic Capacity of WIN-34B, a New Herbal Agent, in the Collagenase-Induced Osteoarthritis Rabbit Model and in Progenitor Cells from Subchondral Bone

**DOI:** 10.1155/2013/527561

**Published:** 2013-08-01

**Authors:** Jeong-Eun Huh, Yeon-Cheol Park, Byung-Kwan Seo, Jae-Dong Lee, Yong-Hyeon Baek, Do-Young Choi, Dong-Suk Park

**Affiliations:** ^1^East-West Bone & Joint Research Institute, Kyung Hee University, 149 Sangil-dong, Gangdong-gu, Seoul 134-727, Republic of Korea; ^2^Department of Acupuncture & Moxibustion, Kang Dong Medical Center, Kyung Hee University, 149 Sangil-dong, Gangdong-gu, Seoul 134-727, Republic of Korea; ^3^Department of Acupuncture & Moxibustion, College of Korean Medicine, Kyung Hee University, 1 Hoegidong, Dongdawmungu, Seoul 130-701, Republic of Korea

## Abstract

We sought to determine the cartilage repair capacity of WIN-34B in the collagenase-induced osteoarthritis rabbit model and in progenitor cells from subchondral bone. The cartilage protective effect of WIN-34B was measured by clinical and histological scores, cartilage area, and proteoglycan and collagen contents in the collagenase-induced osteoarthritis rabbit model. The efficacy of chondrogenic differentiation of WIN-34B was assessed by expression of CD105, CD73, type II collagen, and aggrecan *in vivo* and was analyzed by the surface markers of progenitor cells, the mRNA levels of chondrogenic marker genes, and the level of proteoglycan, GAG, and type II collagen *in vitro*. Oral administration of WIN-34B significantly increased cartilage area, and this was associated with the recovery of proteoglycan and collagen content. Moreover, WIN-34B at 200 mg/kg significantly increased the expression of CD105, CD73, type II collagen, and aggrecan compared to the vehicle group. WIN-34B markedly enhanced the chondrogenic differentiation of CD105 and type II collagen in the progenitor cells from subchondral bone. Also, we confirmed that treatment with WIN-34B strongly increased the number of SH-2(CD105) cells and expression type II collagen in subchondral progenitor cells. Moreover, WIN-34B significantly increased proteoglycan, as measured by alcian blue staining; the mRNA level of type II **α**1 collagen, cartilage link protein, and aggrecan; and the inhibition of cartilage matrix molecules, such as GAG and type II collagen, in IL-1**β**-treated progenitor cells. These findings suggest that WIN-34B could be a potential candidate for effective anti-osteoarthritic therapy with cartilage repair as well as cartilage protection via enhancement of chondrogenic differentiation in the collagenase-induced osteoarthritis rabbit model and progenitor cells from subchondral bone.

## 1. Introduction

Osteoarthritis (OA) is a degenerative joint disease characterized by degradation and loss of articular cartilage, hypertrophic bone changes with osteophyte formation, and subchondral sclerosis [1, 2]. The disease results from homeostatic imbalance between matrix synthesis and degradation of joint tissue [3]. 

Currently, the subchondral bone is recognized as a key factor in normal joint protection and chondrogenic differentiation. Subchondral bone has been shown to exert important shock absorbing and supportive function. Subchondral bone can decrease the joint load and supplies nutrients to cartilage. Moreover, researchers believe that factors produced locally by subchondral bone tissue seep through the bone-cartilage interface [4]. These changes then lead to reactivation of the secondary ossification centre and a decrease in cartilage thickness [5]. Cartilage defects that extend to the subchondral bone exhibit some ability to repair via the formation of neocartilage [6], probably due to the release of bone marrow-derived stem cells from the underlying subchondral bone [7]. 

OA has traditionally been seen as a primary articular cartilage disorder. However, recent observations have demonstrated that both early-stage increased remodeling and bone loss, and late-stage slow remodeling and subchondral densification are important components of the OA pathogenesis [8]. In addition, subchondral bone is thought to play a key role in OA pathogenesis. Subchondral bone changes in OA are potentially both a result and a cause of cartilage loss. Subchondral bone stiffness may decrease its viscoelastic properties and produce a loss of shock absorbing capacity, which in turn causes significant mechanical load and breakdown of the overlying cartilage [9]. Cartilage damage may in turn negatively influence the subchondral bone, thus perpetuating a pathogenic circle in the OA joint. Consequently, modulation of subchondral bone remodeling may become an attractive approach for OA treatment.

Current strategies for OA treatment include decreasing joint pain and stiffness, improving joint function, and delaying surgery. Commonly prescribed OA medications include nonsteroidal anti-inflammatory drugs, analgesics, locally administered corticosteroids, and viscosupplementation, which provide only symptomatic relief and eventually result in the need for surgical intervention [10]. Until now, effective agents have not been found that significantly prevent progression of disease and aid recovery of tissue damage. The search continues for reliable therapeutic agents that influence subchondral bone remodeling and establish their potential role as disease-modifying OA drugs (DMOADs). These agents include antiresorptives (estrogens, selective estrogen receptor modulators, bisphosphonates, calcitonins, and osteoprotegerin and blocking RANKL antibodies), bone-forming agents (parathormone and teriparatide), and antiosteoporotic agents with dual mechanism of action (strontium ranelate) [11]. However, there are still no DMOADs currently available to patients that can prevent disease progression and reverse the damage caused by OA [12]. 

To develop a novel antiosteoarthritis drug, we investigated the cartilage protection, analgesia, and anti-inflammation properties of 200 medicinal herbs used clinically for their anti-inflammatory and analgesic properties in traditional medicine. WIN-34B, a compound extracted from two herbs, the flowers of *Lonicera japonica *Thunb and roots of *Anemarrhena asphodeloides *BUNGE, was initially isolated through a comprehensive screening process. We standardized WIN-34B for quality control according to a previous report [13] and analyzed the major compounds of WIN-34B with the aim of standardizing its practical use and aiding in medicinal development. Data from several previous studies indicate that WIN-34B exhibits excellent analgesic and anti-inflammatory properties in the experimental models [13] and did not cause toxicity or gastric injury when orally administered to rats [14]. Also, WIN-34B showed that anti-inflammatory effect is mediate by reducing inflammatory mediators and regulating MMPs, ADAMTSs, and TIMPs via I*κ*B-*α* and MAP kinases signal pathways in IL-1*β*-stimulated human OA fibroblast-like synoviocytes [15]. Furthermore, WIN-34B has cartilage protective effects in osteoarthritis human cartilage explants culture and chondrocytes [16]. These results suggest that WIN-34B could be a potential candidate for effective antiosteoarthritic therapy with cartilage protective properties and without toxicity instead of existing OA treatment. However, little is known about the effects of WIN-34B on chondrogenic differentiation. 

In this study, we investigated the effects of WIN-34B on cartilage protection and chondrogenic differentiation in the collagenase-induced osteoarthritis rabbit model and progenitor cells from subchondral bone.

## 2. Materials and Methods

### 2.1. Preparation of WIN-34B Extract and Standardization

The dried flowers of *Lonicera japonica* and the dried root of *Anemarrhena asphodeloides* from Song Lim Pharmaceutical Company (Seoul, Republic of Korea) were purchased and identified by the Korea Pharmaceutical Trading Association (Seoul, Republic of Korea). Voucher specimens of *Lonicera japonica* Thunb. (no. OA-LOJ-15) and *Anemarrhena asphodeloides* Bunge (no. OA-ANA-11) were analyzed by HPLC analysis and deposited in the Central Research Institute, WhanIn Pharm. Co. Ltd. (Suwon, Republic of Korea).

WIN-34B was prepared by extracting a mixture of 2 kg of dried *Lonicera japonica* flowers and 1 kg of *Anemarrhena asphodeloides* root (2 : 1, w/w) with 10 L of 50% (v/v) ethanol for 4 h at 85°C. After the extracted solution was filtered and evaporated *in vacuo*, the resulting concentrate was dissolved in 225 mL distilled water and partitioned with 195 mL *n*-butanol. The *n*-butanol layer was evaporated *in vacuo* and lyophilized for complete removal of the residual solvent, resulting in a 7% yield of 11 g brown powder. We standardized WIN-34B for quality control according to a previous report [13], which we then analyzed by HPLC to find the standard compounds, mangiferin and chlorogenic acid.

### 2.2. *In Vivo* Study

#### 2.2.1. Animals

Male New Zealand white rabbits (2.8–3.0 kg, nine to ten weeks) were obtained from the animal experimental center at Kyung Hee University Hospital (Seoul, Republic of Korea) and individually housed with water and food available ad libitum. The room was light/dark (08:00–20:00 h light, 20:00–08:00 h dark) controlled and kept at 21–24°C. All experiments were conducted according to the “Guiding Principles for the Care and Use of Laboratory Animals” and all procedures approved by the Animal Care and Use Committee of Kyung Hee University Medical Center.

#### 2.2.2. Induction of Collagenase-Induced Osteoarthritis and Drug Treatment

Rabbits aged nine to ten weeks and weighing 2.8–3.0 kg at the start of the experiment (day 1) were anesthetized with an intramuscular injection of 0.5 mg/kg tiletamine-zolazepam (Zoletil50, Virbac, France). The shaved right knee joints of all rabbits were intra-articularly injected with either 250 *μ*L of 4 mg/mL collagenase solution (Clostridium histolyticum type II, 425 units/mL enzyme activity) or saline (control group). The same collagenase injection procedure was applied once more on day 4 according to methods described in Mankin [17]. Following the initial injection of collagenase (day 1), the rabbits were divided into groups (*n* = 10 per group). For four weeks, the vehicle groups were orally treated with 20 mL distilled water and the experimental groups were orally treated with WIN-34B (100, 200, and 400 mg/kg), joins (ETCP, SK chemicals, 400 mg/kg), celebrex (CEL, Pfizer, 100 mg/kg), and glucosamine (Gluco-Hcl, Sigma, 1500 mg/kg) on a daily basis using a feeding catheter (DJ2-284, Dae jong Ins. Korea).

#### 2.2.3. Macroscopic Scoring of Stiffness

Stiffness was classified by movement, swelling, and reddening of knees. Each characteristic was subclassified as mild, moderate, and severe compared to the control group. The examination was performed by two independent observers who were blinded to the treatment groups. 

#### 2.2.4. Quantification of Global Histologic Score

After four weeks, the rabbits were sacrificed for histological examination. The right knee joints were then dissected and fixed in 10% phosphate-buffered formalin for two days, then decalcified in Calci-Clear Rapid solution (National Diagnostics, Atlanta, USA) for ten days, and then embedded in paraffin. Standard frontal sections of 5 *μ*m were stained with hematoxylin and eosin (H&E) in the cartilage. Cartilage degradation features were analyzed using the scoring system developed by Colombo et al. [18] and modified by Kikuchi et al. [19]. The following six parameters were quantified: loss of superficial layer, erosion of cartilage, fibrillation and/or fissures, loss of stainable proteoglycan, disorganization of chondrocytes, and loss of chondrocytes. Each item was first graded from 1 to 4 (minimum to maximum damage) and the global histologic score was calculated as the total sum of each of the six parameters. All measurements were performed on the medial part of the tibial plateau, which is the region most affected by collagenase, and the femur condyle in the knee joint.

#### 2.2.5. Quantification of Cartilage Area

All specimens were analyzed under bright-field microscopy (Axiovert 200, Carl Zeiss, Germany), and images were captured using a CCD camera (Axiocam MRc5, Carl Zeiss, Germany). The area of cartilage was measured in the whole of cartilage in the knee joints (100x magnification). All sections were evaluated by two independent observers blinded to the treatment groups. The results of these evaluations were then statistically analyzed.

#### 2.2.6. Quantification of Proteoglycan by Safranin O Staining and Collagen by Masson's Trichrome Staining 

These sections were also stained with Safranin O for detection of proteoglycan loss and Masson's trichrome for measurement of collagen in the cartilage. Cartilage depletion was indicated visually by diminished Safranin O staining and Masson's trichrome staining and was measured automatically with a Biocom microscope and AxioCam camera (Carl Zeiss, Germany). The proteoglycan content or collagen ratio of the cartilage was approximated by quantifying the staining intensity of the histologic sections [20, 21]. Intensity was measured in both the superficial and deep zone of the cartilage (100x magnification), and the ratio was calculated by dividing the Safranin O staining intensity (SOI) and the Masson's trichrome staining intensity (MTI) in the superficial zone by the SOI and MTI in the deep zone. All sections were evaluated by two independent observers blinded to the treatment groups. The results of these evaluations were then statistically analyzed.

#### 2.2.7. Immunohistochemical Staining

Deparaffinized sections were pretreated with chondroitinase ABC (1 U/mL; SIGMA, St. Louis, MO, USA) at 37°C for 30 min. The endogenous peroxidase was blocked with 3% H_2_O_2_ in PBS at room temperature for 20 min and incubated in normal goat serum at room temperature for 30 min. Immunodetection of type II collagen was performed by raising a new polyclonal rabbit antibody (5 *μ*g/mL; MP Biomedicals Inc., CA, USA) against the six amino-acid sequence (EKGPDP) of the C telopeptide of type II collagen at 4°C overnight. Rabbit anti-aggrecan antibody (10 *μ*g/mL; Affinity BioReagents, Golden, CO, USA) was used to evaluate the aggrecan at 4°C overnight. Rabbit anti-CD105 antibody and rabbit anti-CD73 antibody (Santa Cruz Biotechnology, CA, USA) were treated to detect mesenchymal stem cells. After reaction of the biotinylated rabbit antibodies against goat immunoglobulin G (IgG) (DAKO, Glostrup, Denmark) at room temperature for 30 min, the sections were reacted with horseradish peroxidase-labeled streptavidin (DAKO) at room temperature for 15 min. The color was developed with 3,3-diaminobenzidine tetrahydrochloride (DAB) in 50 mmol/L of Tris-HCl, pH 7.6, 0.15 mol/L of NaCl, containing 0.05% Tween. Counterstaining was performed with hematoxylin. As a control, sections were reacted by replacing the first antibodies with nonimmune goat IgG (DAKO) at room temperature for 2 h before the immunostaining. 

### 2.3. *In Vitro* Study

#### 2.3.1. Isolation and Cultivation of Progenitor Cells from Rabbit Subchondral Bone

Subchondral bone was obtained from the knee joint of NewZealand white rabbit. To harvest progenitor cells from rabbit subchondral bone, bone was cut into small fragments, washed with phosphate buffered saline, and partially digested for 4 h at 37°C using 256 U/mL type I collagenase (Sigma-Aldrich, St Louis, MO, USA). The supernatant was discarded and the remaining fragments were placed in culture flasks and cultured in DMEM medium (Gibco-BRL, now part of Invitrogen Corporation, Carlsbad, CA, USA) containing 10% heat-inactivated FBS and an antibiotic mixture (100 units/mL penicillin base and 100 *μ*g/mL streptomycin) at 37°C in the humidified atmosphere. This medium was replaced every 2 days until cells were observed in the Petri dishes. At this point, the culture medium was replaced with fresh medium containing 10% FBS until confluence. Cells that reached 80%–90% confluence were passaged using trypsin-EDTA in PBS (0.05% v/v, Gibco-BRL) and expanded into plates as passage 1. Medium was exchanged every 3 days.

#### 2.3.2. Flow Cytometry Analysis of Progenitor Cells and Chondrogenic Differentiation of Progenitor Cells from Rabbit Subchondral Bone

Mesenchymal progenitor cells from passage 2 of subchondral bone were washed in PBS/0.2% BSA and stained with fluorescence-isothiocyanate- (FITC-) conjugated mouse anti-human CD73, CD90, and CD45 (BD Biosciences, San Diego, CA, USA) phycoerythrin- (PE-) conjugated anti-human CD105(SH-2), the activated leukocyte cell adhesion molecule (ALCAM, CD166), CD34, and CD11b (BD Biosciences, San Diego, CA, USA) for 15 min on ice. For indirect staining, cells were incubated 30 min with either mouse anti-human STRO-1 or rabbit anti-human type II collagen (Col II)-IgG, washed with PBS/0.2% BSA, and stained with FITC-conjugated anti-mouse IgG (DAKO, Hamburg, Germany) for STOR-1 and PE-conjugated anti-rabbit IgG for Col II for 30 min at 4°C. 

Mesenchymal progenitor cells from passage 2 of subchondral bone were seeded in 6-well plates at a concentration of 5 × 10^5^cells/cm^2^. After 24 h, cells were treated or nontreated with WIN-34B (10 *μ*g/mL) and cultured for 7 days and 14 days. Cells were stained with CD45 FITC/CD105 PE or CD45 FITC/Col II PE for 30 min at 4°C. Cells were analyzed on FACScan (BD Biosciences, San Diego, CA, USA) using Cell Quest software (BD Biosciences San Diego, CA, USA). 

#### 2.3.3. Induction of Chondrogenic Differentiation in IL-1*β*-Stimulated Progenitor Cells of Rabbit Subchondral Bone

Mesenchymal progenitor cells from passage 2 were trypsinized (passage 3), and 2 × 10^5^ cells in 1 mL standard media were centrifuged at 1000 rpm in 15 mL polypropylene. After overnight culture, media was changed to chondrogenic induction media. Chondrogenesis was induced by chondrogenic medium (high glucose DMEM medium supplemented with antibiotic mixture, 1% FBS, 50 *μ*g/mL ascorbic acid-2-phosphate, 0.35 mM proline, 1% ITS^+^, 10 ng/mL transforming growth factor-*β*3 (TGF-*β*3) [22]. Pellet of cells were treated with 5 ng/mL IL-1*β* (R&D Systems, Minneapolis, USA) in the absence or presence for 1 h and then added WIN-34B (1, 10 and 20 *μ*g/mL). The medium was changed three times per week, and cells were maintained for up to 7 days. 

(1) *Histological Analysis of Alcian Blue Staining.* Chondrogenic differentiation was histologically assessed by embedding micromasses in OCT compound and freezing, and cryosectioning at a thickness of 7 *μ*m. Sections were stained with alcian blue, pH 1.0 (Rowley Biochemical, Danvers, MA, USA) to show proteoglycans. 

(2) *Colorimetric Analysis of GAG and Type II Collagen.* GAG levels in the culture medium at seven days from onset of culture were determined by the amount of polyanionic material reacting with 1, 9-dimethylmethylene blue. Twenty microliter samples were mixed with 100 *μ*L of DMB reagents (48 mg/mL DMB, 40 mM glycine, 40 mM NaCl, 10 mM HCl, pH 3.0) for 30 min at room temperature and quantified by measuring the absorbance at 590 nm (Spectramax, Molecular Devices, Sunnyvale, CA, USA). All measurements were performed in quadruplicate. Quantification was performed using a standard curve of chondroitin 6-sulfate from shark cartilage (Sigma) in the range of 0–35 *μ*g/mL. Type II collagen levels were determined using the Sircol Collagen Assay (Biocolor Ltd., Valley Business Center, Northern Ireland). The culture medium at seven days from onset of culture was reacted with Sirius red dye containing sulfonic acid for 30 min at room temperature. The amount of type II collagen was calculated by measuring the absorbance at 540 nm and comparing that value to the standard concentration curve (0–200 *μ*g/mL).

(3) *Analysis of Real-Time Quantitative Reverse Transcription-Polymerase Chain Reaction (qRT-PCR).* Total RNA was isolated using TRIzol reagent according to the manufacturer's protocol. Reverse transcription was performed by M-MLV Reverse Transcriptase (TaKaRa Biotechnology) according to the manufacturer's specifications. Briefly, first-strand cDNA was synthesized at 37°C for 1 h in 20 *μ*L reaction mixture using 1 *μ*g isolated mRNA. Real-time PCR (qRT-PCR) was carried out in a 25 *μ*L volume container with SYBR Green PCR Master Mix (Roche Diagnostics). The template source was either 5 ng cDNA or purified DNA standard. The following primer sequences were used to amplify the type II *α*1 collagen, type I *α*1 collagen, cartilage link protein, and aggrecan (Table 1). *β*-actin was amplified as an internal control to standardize mRNA levels. Relative expression of the target genes in the study samples was obtained using the difference as calculated by the comparative threshold (*C*_*t*_) method. The cycle of threshold (*C*_*T*_) for each sample was averaged and normalized to GAPDH. The results were then analyzed by comparative ΔΔ*C*_*T*_ method (2^(−ΔΔ*C*_*T*_)^) for relative quantification of gene expression.

### 2.4. Statistical Analysis

Data were expressed as mean ± SEM Differences among groups were analyzed by one-way ANOVA. In the case of two groups, a Student's *t*-test was used. Statistical significance was assessed at *P* < 0.05.

## 3. Results

### 3.1. Effects of WIN-34B on Stiffness and Cartilage Loss in the Collagenase-Induced Osteoarthritis Rabbit Model

**Figure 1:**
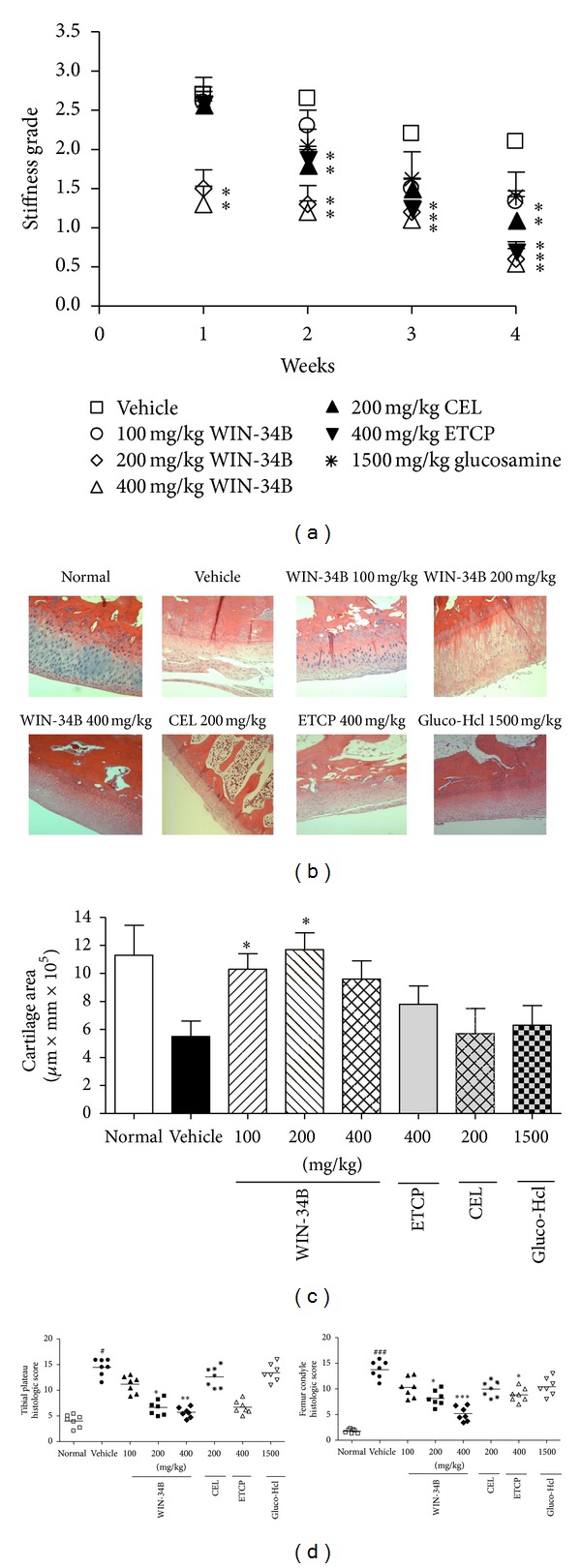
Effects of WIN-34B on disease progression in the collagenase-induced arthritis rabbit model. Right knees of rabbits were intra-articularly injected with collagenase on days 1 and 4. 100 mg/kg, 200 mg/kg, and 400 mg/kg WIN-34B; 200 mg/kg CEL; 400 mg/kg ETCP or 1500 mg/kg Gluco-Hcl were orally administered for four weeks. (a) Stiffness (b) Hematoxylin-Eosin stained sections of knee joints from the normal, vehicle, WIN-34B 100 mg/kg, WIN-34B 200 mg/kg, WIN-34B 400 mg/kg, CEL 200 mg/kg, ETCP 400 mg/kg, and Gluco-Hcl 1500 mg/kg. (c) Cartilage area and (d) histopathologic scores evaluated at the tibial plateau and femur condyle in animals with collagenase-induced arthritis. Values are the mean ± SEM. ^#^ *P* < 0.05 and ^###^ *P* < 0.001 compared to the normal group, *P < 0.05, **P < 0.01, and ***P < 0.001 compared to the vehicle group. Two independent experiments were performed with similar results.

Administration of WIN-34B dose dependently reduced knee stiffness in one week after treatment compared to the vehicle. In contrast, there was no significant improvement of stiffness in the CEL, ETCP, and Gluco-Hcl (Figure 1(a)). After three weeks, CEL at 200 mg/kg and ETCP at 400 mg/kg reduced these symptoms when compared to the vehicle. WIN-34B exhibited some mild changes such as structure or chondrocyte loss (Figure 1(b)). Morever, WIN-34B at 100, 200, and 400 mg/kg led to 1.8-, 2.0-, and 1.9-fold increases in cartilage area, respectively, compared to vehicle (Figure 1(c)). WIN-34B at 200 and 400 mg/kg significantly decreased cartilage degradation of the tibial plateau (2.3- and 2.4-fold) and femur condyle (1.8- and 2.2-fold), respectively, compared to the vehicle. Moreover, tibial plateau and femur condyle change tended to be higher in the WIN-34B at 200 and 400 mg/kg compared to other experimental groups (CEL, ETCP, and Gluco-Hcl) (Figure 1(d)).

### 3.2. Effects of WIN-34B on Cartilage Protection in the Collagenase-Induced Osteoarthritis Rabbit Model

**Figure 2:**
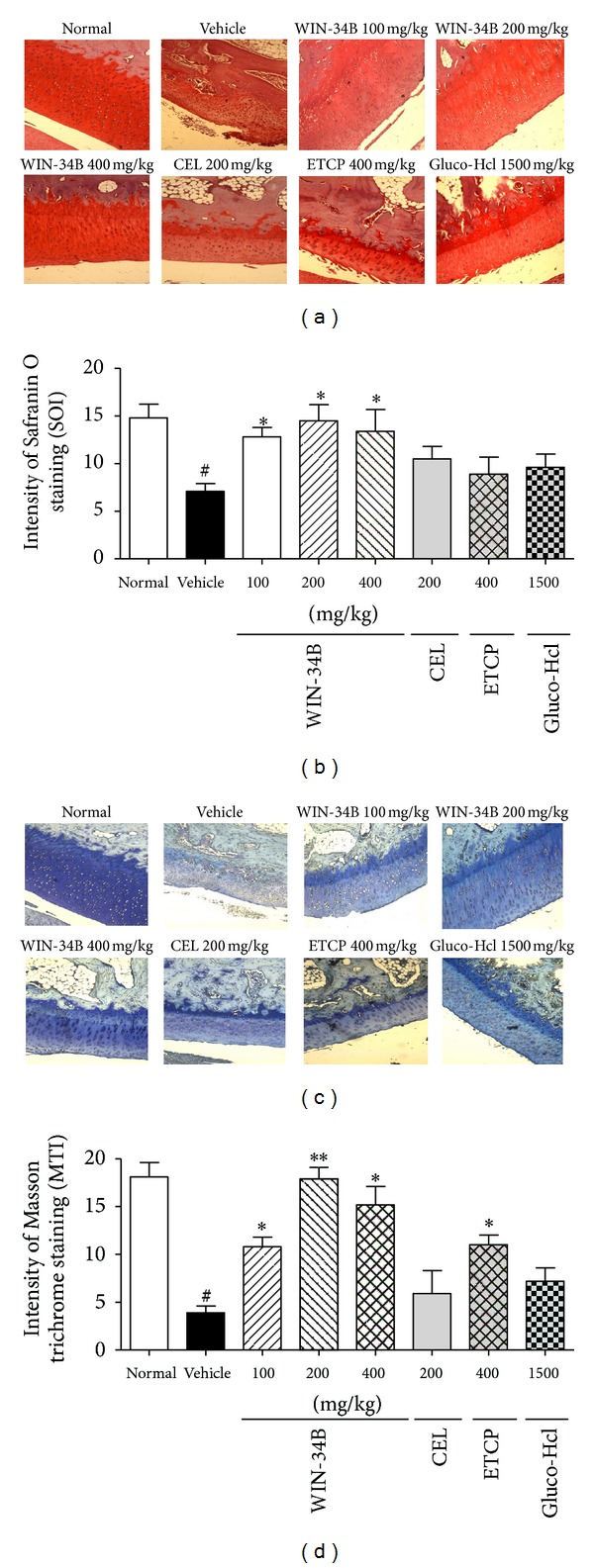
Effects of WIN-34B on cartilage protection in the knees of collagenase-induced arthritis rabbits. (a) Representative Safranin O stained sections of knee joints from the normal, vehicle, WIN-34B 100 mg/kg, WIN-34B 200 mg/kg, WIN-34B 400 mg/kg, CEL 200 mg/kg, ETCP 400 mg/kg, and Gluco-Hcl 1500 mg/kg group. (b) Proteoglycan content, expressed as a ratio, was calculated by dividing the Safranin O staining intensity (SOI) in the superficial zone (SOI-S) by SOI in the deep zone (SOI-D). (c) Representative Masson's trichrome stained sections of knee joints from the normal, vehicle, WIN-34B 100 mg/kg, WIN-34B 200 mg/kg, WIN-34B 400 mg/kg, CEL 200 mg/kg, ETCP 400 mg/kg and Gluco-Hcl 1500 mg/kg group. (d) Collagen content, expressed as a ratio, was calculated by dividing the Masson's trichrome staining intensity (MTI) in the superficial zone (MTI-S) by MTI in the deep zone (MTI-D). Values are mean ± SEM. ^#^ *P* < 0.05 compared to the normal group, *P < 0.05 and **P < 0.01 compared to the vehicle group. Two independent experiments were performed with similar results.

Cartilage protective effects of WIN-34B were also evaluated by Safranin O staining for detection of proteoglycan loss and Masson's trichrome staining for measurement of collagen in the cartilage. In the normal, proteoglycan and collagen contents preserved in the knee joints, but not resulting in a marked loss of proteoglycan and collagen in the collagenase-induced knee joints (Figures 2(a) and 2(c)). WIN-34B at 100, 200, and 400 mg/kg increased the ratio of SOI (1.8-, 2.1-, and 2.0- fold) and MTI (2.2-, 4.2-, and 3.7-fold), respectively, compared to the vehicle. This ratio was also significantly higher in the WIN-34B at 100, 200, and 400 mg/kg compared to other experimental groups (CEL, ETCP, and Glugo-Hcl) (Figures 2(b) and 2(d)).

### 3.3. Effects of WIN-34B on Chondrogenic Differentiation in the Collagenase-Induced Osteoarthritis Rabbit Model

**Figure 3:**
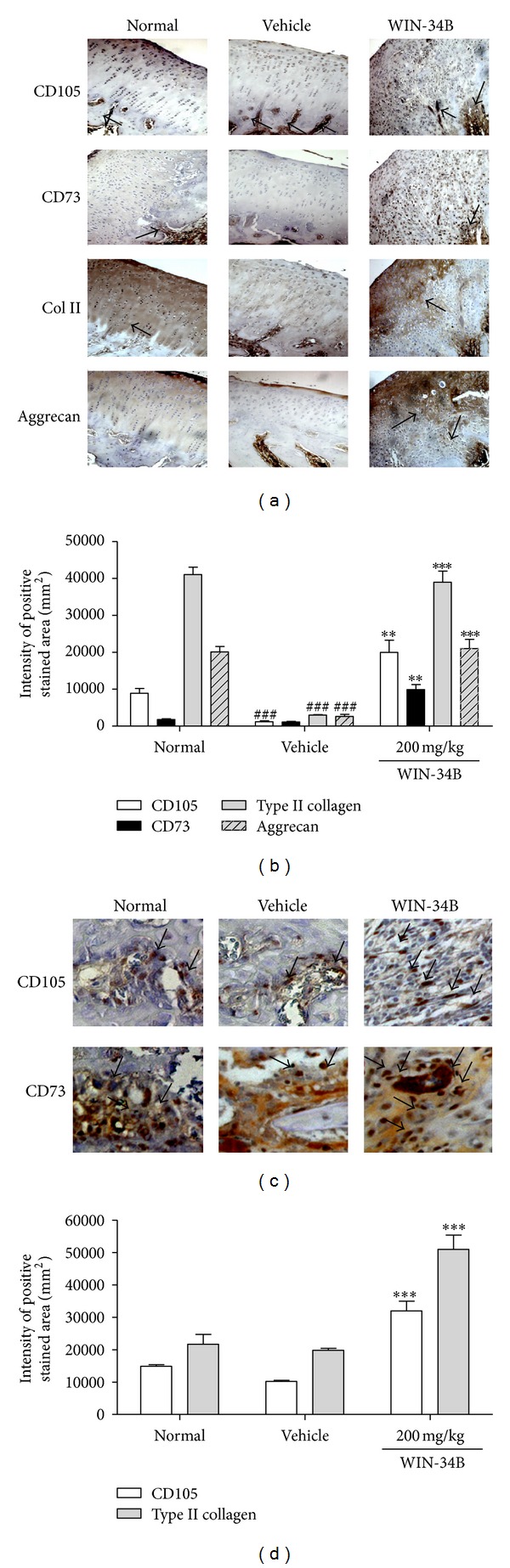
Effects of WIN-34B on chondrogenic differentiation in the knees of collagenase-induced arthritis rabbits. (a) Representative immunostained stained sections of cartilage from knee joints of animal in the normal, vehicle, and WIN-34B 200 mg/kg group. Lane 1: rabbit anti-CD105 antibody stained sections, lane 2: rabbit anti-CD73 antibody stained sections, lane 3: rabbit-anti-type II collagen (Col II) antibody stained sections, lane 4: rabbit-anti-aggrecan antibody stained sections. (b) Intensity of positive stained area of anti-CD105, anti-CD73, anti-type II collagen, and anti-aggrecan by immunostaining in the cartilage zone of knee joints. (c) Representative immunostained stained sections of subchondral bone from the knee joints of animals in the normal, vehicle, and WIN-34B 200 mg/kg groups. Lane 1: rabbit anti-CD105 antibody stained sections, lane 2: rabbit anti-CD73 antibody stained sections. (d) Intensity of positive stained area of anti-CD105 and anti-CD73 in the subchondral bone zone of knee joints. Values are the mean ± SEM. ^##^ *P* < 0.01 compared to the normal group, **P < 0.05 and ***P < 0.01 compared to the vehicle group. Two independent experiments were performed with similar results.

According to immunohistochemical analysis, WIN-34B at 200 mg/kg enhanced the expression of positive cells for CD105 (16.6-fold), CD73 (9.0-fold), type II collagen (13.1-fold), and aggrecan (7.9-fold) compared to cartilage area of vehicle (Figures 3(a) and 3(b)). Also, treatment with WIN-34B at 200 mg/kg enhanced the expression of CD105 (2.6-fold) and CD73 (2.7-fold) in the subchondral bone zone (Figures 3(c) and 3(d)).

### 3.4. Effects of WIN-34B on Chondrogenic Differentiation of Progenitor Cells from Rabbit Subchondral Bone

**Figure 4:**
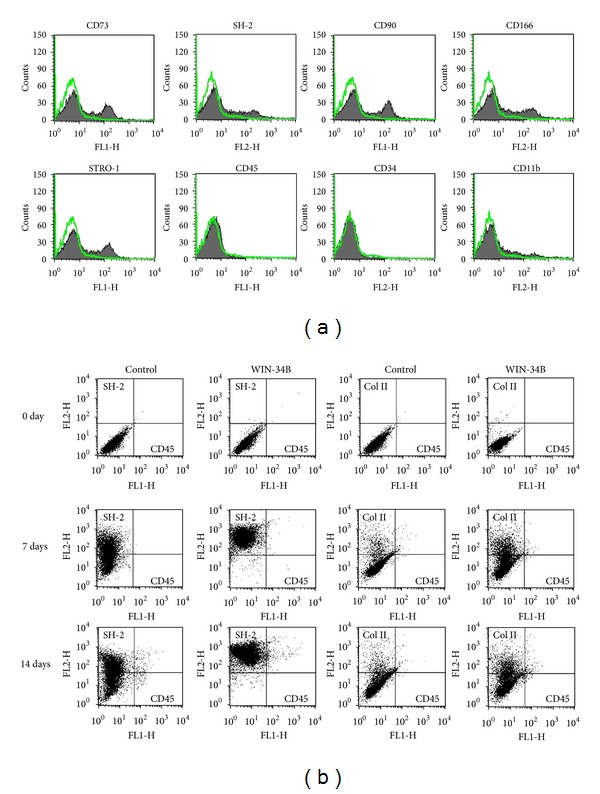
Effects of WIN-34B on chondrogenic differentiation of progenitor cells from rabbit subchondral bone. (a) Surface marker profiling of progenitor cells using flow cytometry. Cells were positive for CD73, SH-2, CD90, CD166, and STOR-1, and negative for CD45, CD34, and CD11b. (b) Chondrogenic induction of progenitor cells from subchondral bone cultured by WIN-34B treatment in chondrogenic medium. Progenitor cells from passage 2 of subchondral bone were seeded in 6-well plates at a concentration of 5 × 10^5^cells/cm^2^. After 24 h, cells were treated or nontreated with WIN-34B (10 *μ*g/mL) and cultured for 7 days and 14 days. Each of the three samples was measured for profiling markers.

**Figure 5:**
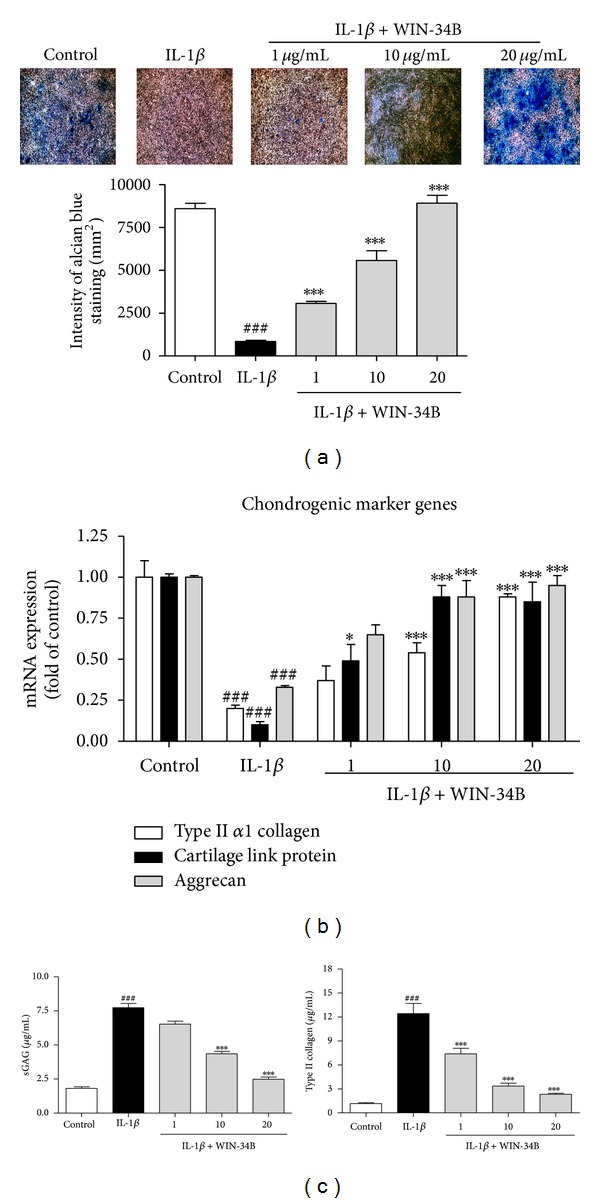
Effects of WIN-34B on chondrogenic differentiation of IL-1*β*-stimulated progenitor cells from rabbit subchondral bone. (a) Histological analysis of WIN-34B by alcian blue staining of chondrogenic differentiation in IL-1*β*-treated progenitor cells. Control, IL-1*β*, WIN-34B 1 *μ*g/mL, WIN-34B 10 *μ*g/mL, and WIN-34B 20 *μ*g/mL after 7 days of culture in chondrogenic differentiation media. Magnified view (×100). (b) Dose response of WIN-34B on the mRNA expression of chondrogenic markers. Chondrogenic differentiation of subchondral progenitor cells that were incubated for seven days with 1, 10, and 20 *μ*g/mL of WIN-34B in the presence of IL-1*β*. qRT-PCR was then performed for type II *α*1 collagen, cartilage link protein, and aggrecan. (c) Inhibitory effects of WIN-34B on GAG and type II collagen degradation on chondrogenic differentiation of IL-1*β*-stimulated subchondral progenitor cells. GAG and type II collagen degradation are shown as a cumulative release into the culture medium. Values are the mean ± SEM. ^###^ *P* < 0.001 compared to the control group. *P < 0.05, ***P < 0.001 compared to the IL-1*β* group. Data were obtained from at least three independent experiments.

Expended progenitor cells showed typical cell surface antigens of mesenchymal stem and progenitor cells (Figure 4(a)). The antigens CD73, SH-2, CD90, and CD166 were present in progenitor cells from rabbit subchondral bone. Cells were positive for CD73 (44%), SH-2 (2–6%), CD90 (39%), CD166 (27%), and STRO-1 (31%). A population of cells were minority positive for SH-2, and negative for the hematopoietic antigen CD34 as well as for the leukocyte common antigen CD45 and macrophage antigen CD11b (Figure 4(a)). We also investigated the *in vitro* effects of WIN-34B on chondrogenic differentiation of progenitor cells derived from rabbit subchondral bone using flow cytometry analysis. The surface antigen for SH-2 was increased by 39% at 7 days and 52% at 14 days of culture in chondrogenic medium, and the expression of type II collagen was enhanced by 8–11% at 7 days and 14 days (Figure 5(b)). WIN-34B at 10 *μ*g/mL increased the number of SH-2 cells (96% and 98%) and expression of type II collagen (23% and 19%) at 7 days and 14 days after cultivation, respectively (Figure 4(b)).

### 3.5. Effects of WIN-34B on Chondrogenic Differentiation of IL-1*β*-Treated Progenitor Cells from Rabbit Subchondral Bone

To evaluate the effects of WIN-34B on the chondrogenic differentiation of IL-1*β*-treated progenitor cells, cells were cultured in high-density pellets of chondrogenic conditions and added WIN-34B at 1, 10, and 20 *μ*g/mL. According to histological analysis, IL-1*β*-treatment of subchondral progenitor cells led to an 8.0-fold reduction in proteoglycan expression compared to the control. However, treatment with WIN-34B at 1, 10, and 20 *μ*g/mL led to 3.2-, 6.0-, and 9.7-fold increases, respectively, compared to the IL-1*β*-treated subchondral progenitor cells (Figure 5(a)). The level of chondrogenic markers was determined by qRT-PCR. IL-1*β* significantly decreased the mRNA expression of type II *α*1 collagen, cartilage link protein, and aggrecan. However, WIN-34B dose dependently increased the level of type II *α*1 collagen, cartilage link, and aggrecan compared to IL-1*β*-treated cells (Figure 5(b)). Moreover, WIN-34B dose dependently reduced the degradation of GAG and type II collagen induced by IL-1*β* compared to IL-1*β*-treated cells (Figure 5(c)). 

## 4. Discussion

In this study, we investigated the effects of WIN-34B on cartilage protection and chondrogenic differentiation in the progress of OA. First, in the collagenase-induced osteoarthritis rabbit model, we evaluated the stiffness and cartilage loss to assess the effects of WIN-34B on disease progression. Cartilage protective effect was confirmed by determining proteoglycan and collagen contents. Chondrogenic capacity of WIN-34B was assessed by the expression of CD105, CD73, type II collagen, and aggrecan using the immunohistochemical analysis. Second, we evaluated the expression of mesenchymal stem cell-related cell surface antigen to assess the chondrogenic induction of progenitor cells from subchondral bone by WIN-34B treatment in chondrogenic medium. The cartilage protective and chondrogenic capacity of WIN-34B was assessed by alcian blue staining of proteoglycan, the mRNA levels of chondrogenic maker genes, and the degradation of GAG and type II collagen in IL-1*β*-treated progenitor cells from rabbit subchondral bone. 

Oral administration of WIN-34B resulted in a significant reduction of general clinical and histological scores. Also, WIN-34B significantly inhibited cartilage loss, as determined by measuring the proteoglycan and collagen. However, histopathology grading showed that CEL and Gluco-Hcl had no protective effect on cartilage, and ETCP resulted in significantly degraded cartilage in the femur condyle, but not the tibial plateau. These results suggest that WIN-34B is markedly more effective against cartilage destruction than the selective COX-2 inhibitor, ETCP, and Gluco-Hcl in the collagenase-induced osteoarthritis rabbit model. Our previous *in vitro* data on WIN-34B has shown cartilage protective effects through the regulation of matrix proteinases (aggrecanases and MMPs/TIMPs), inflammatory mediators (PGE2, NO, IL-1*β*, and TNF-*α*), and the MAPK pathways in osteoarthritis human cartilage explants culture and chondrocytes [15]. These results support the safety and therapeutic usefulness of WIN-34B for development as an OA treatment. 

The ultimate goal of OA treatment is cartilage protection and repair but is not yet clear. Although, various surgical methods have been proposed to regenerate articular cartilage, including bone marrow stimulation [23], mosaicplasty [24], and autologous chondrocyte implantation [25], each of them has some disadvantage such as degeneration of repaired tissue, limitation of large defects, and long-term risk of developing OA [26]. 

There have been many reports of new DMOAD candidates with strong cartilage protective and chondrogenic activities. Antiresorptive agents, such as estrogens, and other bisphosphonates (BP) have been shown to have a chondroprotective function and a favorable effect in stopping OA progression in animal models [27–33]. Recently, selective estrogen receptor modulators (SERMs) have also demonstrated similar positive effects in OA treatment [27]. However, results from clinical trials using these drugs have been contradictory [34]. Bone-forming agents such as PTH (1–34) inhibited the terminal differentiation of human articular chondrocytes *in vitro* and reduced the progression of cartilage damage in a model of papain-induced OA in rats [35]. Strontium ranelate is an agent with dual mechanisms of action on bone metabolism, exerting antiresorptive and new bone forming effects [36]. However, there are few studies on the use of PTH (1–34) and strontium ranelate in animal models of OA, so more research need to be carried out. Therefore, it is reasonable to assume that drugs affecting subchondral bone remodeling may have a prominent role as DMOADs. While various attempts have been made to develop DMORDs, the results have been unsatisfactory regarding the effects and safety of these drugs [37]. 

Progenitor cells of subchondral bone were expected to be an alternative for cartilage repair of OA. Many researchers agree with the importance of subchondral bone healing in the surgical and drug treatment of OA [38, 39]. Studies have attempted to find an effective combination of antigens in order to isolate a pure population of mesenchymal progenitor cells from various tissues [40]. One of these studies found that the coexpression of CD105 and CD73 could be sufficient in the adult human bone marrow cells [41]. Recently, micromass culture has been widely used to evaluate the chondrogenic potential of mesenchymal progenitor cells. Mesenchymal progenitor cells from human bone marrow increased the expression of type II and type X collagen in micromass culture [42]. Several studies reported that progenitor cells of human subchondral bone promoted the chondrogenic maker genes such as type II collagen, aggrecan, cartilage link protein, and cartilage oligomeric matrix protein [43–45]. In our study, WIN-34B increased the significant expression of CD105 and CD73 in the subchondral bone of collagenase-induced osteoarthritis rabbit model. Moreover, type II collagen and aggrecan fully recovered from damage by WIN-34B at 200 mg/kg. WIN-34B at 10 *μ*g/mL increased the number of SH-2 cells and expression of type II collagen in subchondral progenitor cells. Furthermore, WIN-34B strongly induced proteoglycan, type II *α*1 collagen, cartilage link protein, and aggrecan and reduced the degradation of GAG and type II collagen in the IL-1*β*-treated subchondral progenitor cells. Therefore, we suggest that WIN-34B strongly induced chondrogenic differentiation by the induction of typical mesenchymal stem cell related cell surface antigen CD105 and CD73, and the enhancement of chondrogenic markers type II collagen and aggrecan *in vivo *and *in vitro*. 

 In summary, it was shown that WIN-34B exerted cartilage protection and chondrogenic differentiation in the collagenase-induced osteoarthritis rabbit model and progenitor cells from subchondral bone by stimulating chondrogenic differentiation of progenitor cells derived from subchondral bone. These results suggest that WIN-34B has potential for a new disease-modifying osteoarthritis drug (DMOAD) candidate in OA treatment. 

## Figures and Tables

**Table 1:** Primers of targeted genes.

mRNA	Primers	Annealing Tm (cycle)
Type II *α*1 collagen	Fw: 5′-AAC ACT GCC AAC GTC CAG AT-3′	58°C (32)
	Rv: 5′-CTG CAG CAC GGT ATA GGT GA-3′	

Type I *α*1 collagen	Fw: 5′-TGA CCT CAA GAT GTG CCA CT-3′	58°C (32)
	Rv: 5′-GGG AGT TTC CAT GAA GCC-3′	

Cartilage link protein	Fw: 5′-GCG TCC GCT ACC CCA TCT CTA-3′	55°C (32)
	Rv: 5′-CTC TAA GGG CAC ATT CAC TT-3′	

Aggrecan	Fw: 5′-GAG GTC GTG GTG AAA GGT GT-3′	58°C (32)
	Rv: 5′-GTG TGG ATG GGG TAC CTG AC-3′	

GAPDH	Fw: 5′-GCT CTC CAG AAC ATC ACT CCT GCC-3′	58°C (30)
	Rv: 5′-CGT TGT CAT ACC AGG AAA TGA GCT T-3′	

Fw: forward; Rv: reverse; GAPDH: glyceraldehyde-3-phosphate dehydrogenase Tm: temperature.
